# CYP11B1 has no role in mitotane action and metabolism in adrenocortical carcinoma cells

**DOI:** 10.1371/journal.pone.0196931

**Published:** 2018-05-07

**Authors:** Antonina Germano, Laura Saba, Silvia De Francia, Ida Rapa, Paola Perotti, Alfredo Berruti, Marco Volante, Massimo Terzolo

**Affiliations:** 1 Internal Medicine, Department of Clinical and Biological Sciences at San Luigi Hospital, University of Turin, Orbassano, Turin, Italy; 2 Pathology, Department of Oncology at San Luigi Hospital, University of Turin, Orbassano, Turin, Italy; 3 Oncology Unit, Department of Surgery, Radiology and Public Health, University of Brescia, ASST Spedali Civili di Brescia, Brescia, Italy; Chapman University, UNITED STATES

## Abstract

Mitotane is the reference drug for adrenocortical carcinoma (ACC) and the metabolic activation of the drug is considered as essential for its activity. The aim of this study was to assess the role of CYP11B1 on mitotane action and metabolism in H295R ACC cells to understand whether this enzyme may influence mitotane action. The simultaneous incubation with mitotane and metyrapone, an adrenolytic molecule targeting 11-beta-hydroxylase, did not influence mitotane-mediated cytotoxic effect and metabolism in H295R ACC cells. CYP11B1 silencing confirmed the lack of a significant metyrapone effect on mitotane action. The present findings do not support the view that CYP11B1 catalyzes a crucial step in the metabolic activation of mitotane and that CYP11B1 confers the adrenal specificity to mitotane.

## Introduction

Adrenocortical carcinoma (ACC) is a rare and malignant endocrine tumor with a generally poor prognosis [1, 2]. Mitotane (o,p’DDD), a metabolite of the insecticide dichlorodiphenyltrichloroethane (DDT), is the reference treatment for ACC, and is used either in the post-operative adjuvant setting following ACC extirpation or for treatment of advanced ACC [3, 4]. Mitotane acts on the adrenal glands inhibiting cell growth and impairing steroidogenesis [5]. Critical steps of the inhibitory effects on steroidogenesis may occur in mitochondria possibly involving CYP11A1, a mitochondrial enzyme that catalyzes the transformation of cholesterol to pregnenolone [6]. Elevated levels of 11-deoxycortisol and 11-deoxycorticosterone in mitotane-treated patients suggest that mitotane may affect CYP11B1 [7].

Metabolic activation of mitotane is considered to be essential for the therapeutic effect, through transformation into two metabolites, 1,1-(o,p’-dichlorodiphenyl)-2,2 dichloroethene (o,p’-DDE) and 1,1-(o,p’-dichlorodiphenyl) acetic acid (o,p’-DDA) by α- and β-hydroxylation, respectively [8]. Mitotane is metabolized through a reactive acyl chloride thought to bind adrenal cortical bionucleophiles as well as to serve as the intermediate in the formation of o,p’-DDA, probably by means of a cytochrome P450 (CYP) enzyme [9, 10]. The metabolic activation of mitotane may take place in the tumor and be dependent on either CYP11B1 or a non-steroidogenic CYP, and there is some evidence that the capability of the tumor to metabolize mitotane may predict response to treatment [11, 12]. Although there is no straight evidence that CYP11B1 is the key CYP enzyme in this process, there are in vitro and ex vivo data providing indirect support to the view that CYP11B1 activates mitotane [13, 14]. Transfection of the CYP11B1 gene into monkey COS cells was able to induce uptake and metabolization of a mitotane analog, MeSO2-DDE [15]. It should be taken into account, however, the different molecular structure of MeSO2-DDE compared to mitotane. Overall, the literature on this topic remains controversial [16]. It may seem paradoxical that the activity of mitotane may derive from metabolic transformation via an enzyme system that is inhibited by mitotane itself. However, the effect of mitotane on CYP11B1 mRNA expression was recently reported to be biphasic, being more stimulatory than inhibitory over a wide concentration range of the drug [17].

Metyrapone has been in use since the 1960s for the medical management of Cushing’s syndrome [18]. Metyrapone has been used alone or in combination with other agents including mitotane [19], and explicates its action by inhibiting adrenal beta-hydroxylation of 11-deoxycortisol, the final step in cortisol synthesis. The block of this enzymatic reaction results in a reduction of adrenal cortisol production [20]. Metyrapone did not show any significant interference on o,p′-DDD binding in murine adrenocortical Y-1 cells [21] but partially reduced o,p′-DDD binding in human adrenal tissue-slice culture [14].

Despite that mitotane has been largely used to treat ACC patients, we still do not know precisely the mechanisms of action of this old drug. The aim of this study was to assess the role of CYP11B1 on mitotane action and metabolism in an ACC cell line to understand whether this enzyme may influence mitotane action.

## Materials and methods

### Cell culture and chemical reagents

H295R ACC cell line (ATCC CRL-2128) was supplied from the American Type Culture Collection (ATCC, Rockville, MD, USA). The H295R cells were cultured in a 1:1 mixture of Dulbecco's Modified Eagle's Medium and Ham's F-12 Nutrient mixture (DMEM/F12) (Sigma-Aldrich, Saint Louis, United States, Saint Louis, United States) supplemented with 2 mmol/L L-glutamine, penicillin (25 units/mL), and streptomycin (25 mg/mL, all from Sigma-Aldrich, Saint Louis, United States) and 2.5% of Nu-Serum (Corning, NY,USA) and enriched with 1% di ITS+Premix (Corning, NY,USA). Mitotane (purity >99.9%, Cat. Numb. 49015, Supelco, Bellefonte, United States) was dissolved in 100% methanol (Sigma-Aldrich, Saint Louis, United States) and metyrapone (Sigma-Aldrich, Saint Louis, United States) was dissolved in DMSO.

### Treatment and cell viability assay

H295R cells were seeded into 96-well plates in triplicates and treated with mitotane (from 5 to 50 μM) and metyrapone (20 μM) used alone or in association for 48h. Mitotane concentrations are in a range comparable to that associated with therapeutic effect *in vivo* [9]. Metyrapone concentration is close to the peak concentration of 16.3 μM observed in plasma after 1h following administration of 750 mg of the drug [22]. After incubation time, Cell Proliferation Reagent WST-1 (Roche Applied Science, Penzberg, Germany) was added to each well in order to measure cell viability, following the supplied protocol. The absorbance was determined using a microplate reader (iMARK microplate reader) (Biorad Life Science Group, Hercules, CA USA) at a test wavelength of 450 nm and reference wavelength of 630 nm.

### Measurement of mitotane and its metabolites

Mitotane (o,p’-DDD), o,p’-DDE and o,p’-DDA concentrations were evaluated using a HPLC system (High Pressure Liquid Chromatography, VWR-Hitachi system, LaChrom Elite) after previous specific extraction [23]. These compounds were extracted from equal amounts of cell lysates and surnatants after mitotane treatment (25 μM) for 48h alone or with metyrapone (20 μM) and after CYP11B1 siRna inhibition. Extraction of mitotane, o,p'-DDE and p,p'-DDT, used as internal standard (IS), was performed by vortex mixing of 500 μl of samples with 100 μl of IS (100mg/ml) and 750 μl of acetone. Samples were then centrifuged at 12000 rpm for 5 min and 500 μl of organic layer were transferred to a HPLC vial mixed with 500 μl of recovering phase (HPLC-grade water-methanol-acetonitrile, 40:10:50, v/v/v) for injection. Separation was then achieved with a C18 reverse-phase column (LiChroCART^®^ 250–4 HPLC Cartridge, LiChrospher^®^ 100, RP-18, 5 mm, VWR) preceded by a specific guard column. Chromatographic analysis was carried out at 30°C by a gradient of HPLC-grade water, methanol, acetonitrile (0–6.5 min 10:10:80, v/v/v; 6.6–9.7 min 5:5:90, v/v/v; 9.7–15 min 10:10:80, v/v/v) at the constant flow rate of 1.0 ml/min. The eluate was scanned at 218 nm.

A different method was adopted for o,p'-DDA to eliminate interferences at the compound retention time. Extraction of o,p'-DDA and nilotinib, used as IS, was performed by protein precipitation: 50 μl of IS working solution (200mg/ml) were added to 500 μl of samples. After 750 μl of protein precipitation solution (HPLC-grade water and methanol, 50:50, v/v) were added to each sample, tubes were vortexed for 30 sec and then centrifuged at 12000 rpm for 10 min. Finally 800 μl of organic layer were transferred to a HPLC vial for injection. Separation was then achieved with a C18 reverse-phase column (LiChroCART^®^ 250–4 HPLC Cartridge, LiChrospher^®^ 100, RP-18, 5 mm, VWR) preceded by a specific guard column. Chromatographic analysis was carried out under isocratic elution at 35°C by a mobile phase consisting of 40% solvent A (72.5% water, 25% methanol, 2.5% triethylamine), 20% methanol, and 40% acetonitrile at the constant flow rate of 0.9 ml/min. The eluate was scanned at 267 nm. System management and data acquisition were performed with the EzChrom Elite software. Quantification was performed by IS calibration; a linear regression was used in order to obtain the best fit for all calibration points. Regression coefficient (r^2^) of all calibration curves was higher than 0.99 for all compounds.

### Measurement of cortisol

Cortisol was measured from equal amounts of surnatant after mitotane treatment for 48h alone or with metyrapone and after CYP11B1 siRna inhibition. Cortisol levels were measured on surnatants and were normalized by the number of cells harvested in order to take into account the toxic effects of mitotane. Cortisol was measured by commercially available radioimmunoassay kits (Beckman Coulter, Fullerton, CA, United States). Dynamic ranges and detection limits were as follows: cortisol: 7.2–724 μg/L. Intra- and inter-assay coefficients of variation values were <5%.

### Evaluation of CYP11B1 expression

H295R cells were cultured into six-well plates and treated with mitotane for 48 h. Total RNA was extracted using Qiazol Reagent (Qiagen, Hilden, Germany). Complementary DNA was generated using M-MLVT RT (200U/μl) (Invitrogen, Carlsbad, United States) and oligodT primers (500μl/ml) (Invitrogen, Carlsbad, United States) from 1μg of total RNA. Relative cDNA quantification for CYP11B1 and a housekeeping gene (beta-actin) were examined by quantitative real-time (RT)-PCR using a validated assay probe (RealTime ready Catalog Assays, Roche Applied Science, Penzberg, Germany). To analyze target gene expression in treated cells, levels of CYP11B1 mRNA were normalized to housekeeping gene beta-actin, then ΔΔCt calculation was performed and corresponding values were expressed as 2^-ΔΔCt^.

### Silencing experiments

For CYP11B1 silencing experiments, pre-designed iBoni siRna Pool targeting CYP11B1 and pre-designed iBoni siRna Pool negative control were used (RIboxx, Radebeul, Germany). INTERFERin siRna transfection reagent was purchased from Polyplus transfection (Illkirch, France). Briefly, using a reverse transfection, siRnas (at a final concentration of 50 nmol/L) and INTERFERin were diluted in medium without serum and incubated for 10 minutes at room temperature. Lipoplexes were then transferred to multi-well tissue culture plates and overlaid with H295R ACC cells. After 24 hours, the medium was changed, and the cells were then cultured in medium alone (basal) or in medium containing mitotane (from 5 to 50 μM for cell viability, 25μM for HPLC analysis). Assessment of cell viability, measurement of cortisol, mitotane and its metabolites were done as described above.

### Statistical analysis

Statistical analyses were done by means of the Student’s t-test. All p values reported are the result of two-sided tests. Data are presented as mean ± standard error (SE). Level of statistical significance is <0.05.

## Results

### Effects of metyrapone and mitotane on cortisol production

**Fig 1:**
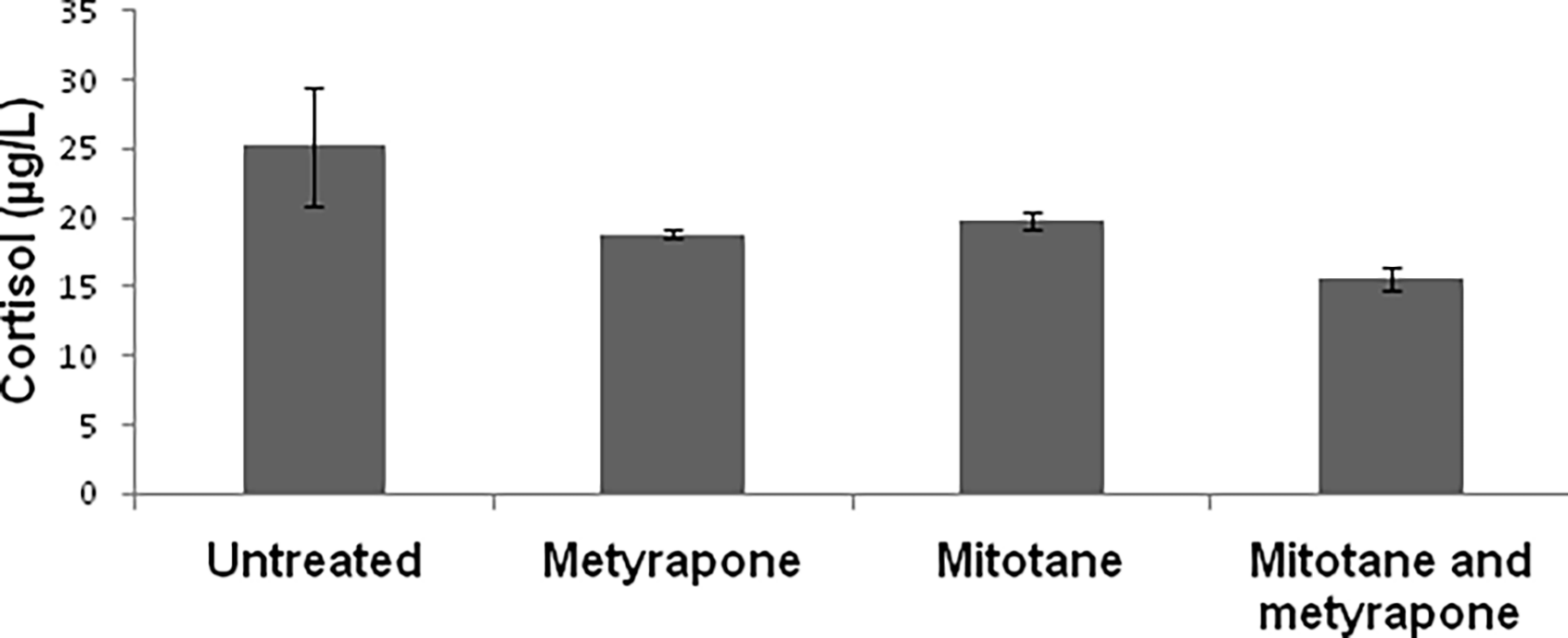
Effects of 25 μM mitotane and 20 μM metyrapone on cortisol levels. Four independent experiments were used to determine each data point. Metyrapone or mitotane treatment vs. untreated cells, P <0.05. Combined treatment with mitotane and metyrapone vs. untreated cells. P = 0.005.

In H295R cells, cortisol levels were significantly reduced after a 48h treatment with metyrapone (20 μM) and mitotane (25 μM), used either alone or in association. Cortisol levels in untreated cells of 25.2 ± 4.3 μg/L were reduced by metyrapone treatment to 18.9 ± 0.3 μg/L (p = 0.03) and by mitotane to 19.8 μg/L ± 0.7 μg/L (p = 0.049). After combined treatment with mitotane plus metyrapone, cortisol levels were 15.6 ± 0.8 μg/L (p = 0.005) (Fig 1) (S1 Table).

### Effects of metyrapone on mitotane activity

**Fig 2:**
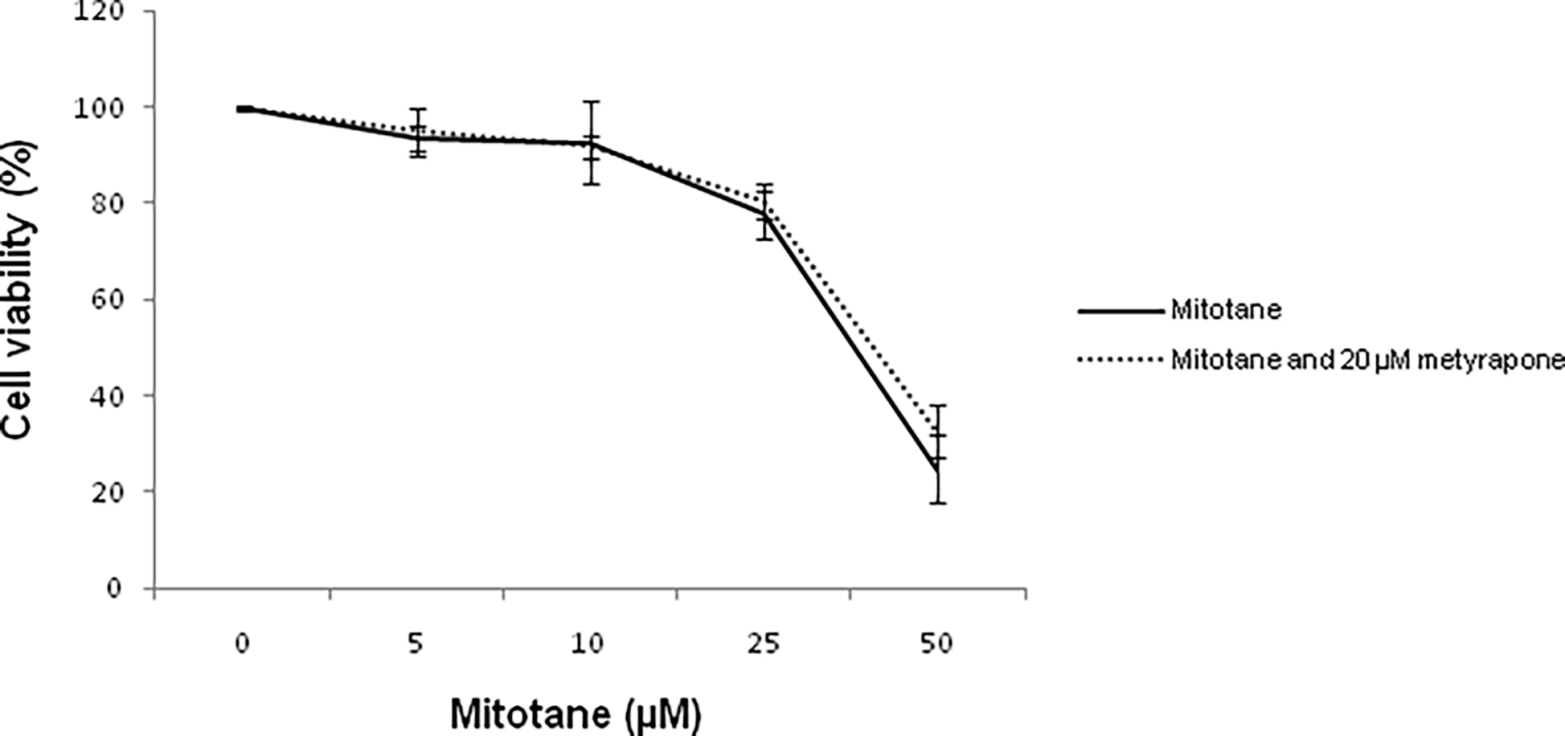
Effects of mitotane and 20 μM metyrapone on H295R cell viability. Three technical replicate wells for each experiment (n = 3) were used to determine each data point. P = ns comparing mitotane treatment vs. combined treatment with mitotane and metyrapone.

**Fig 3:**
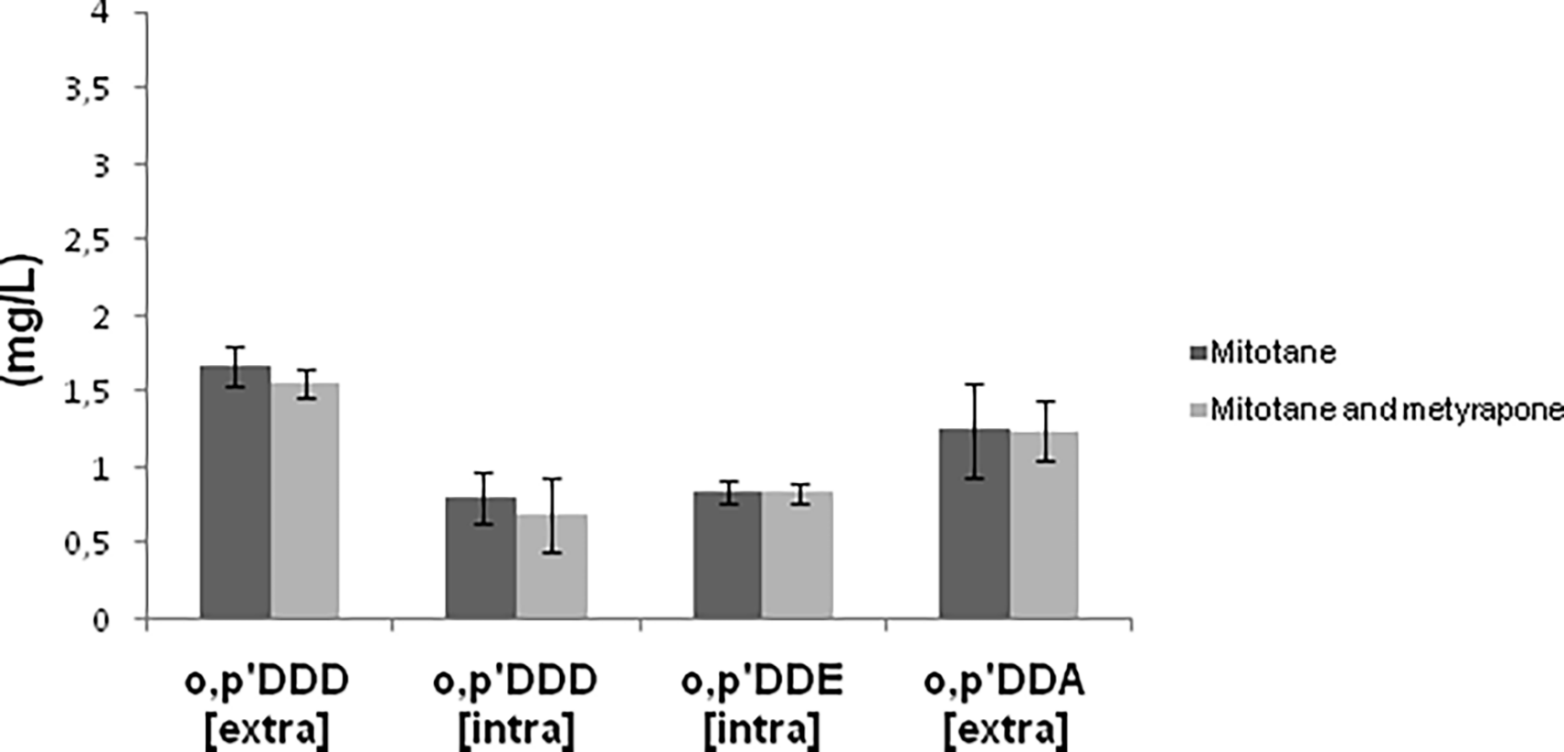
Mitotane metabolites in ACC cells: Uptake, bioavailability and metabolization. Intracellular and extracellular concentration of mitotane and its metabolites in H295R, measured by means of HPLC–UV after metyrapone treatment. Six independent experiments were used to determinate each data point.

Mitotane treatment induced a cytotoxic effect in H295R ACC cell line. The combination of mitotane and metyrapone showed a similar effect on cell viability in comparison to mitotane alone (Fig 2). Metyrapone did not show any effect on cell viability -cell viability ratio for untreated control, 99.8 **±** 0.33 vs 20μM metyrapone, 100.01 **±** 0.25, (p = ns) (S2 Table). Levels of mitotane, o,p’DDE and o,p’DDA were measured in cellular pellets and in surnatants. Their levels in both intracellular and extracellular compartments of H295R cells were not influenced by concomitant treatment with metyrapone (p = ns) (Fig 3) (S3 Table).

### Effects of mitotane on CYP11B1 gene expression

**Fig 4:**
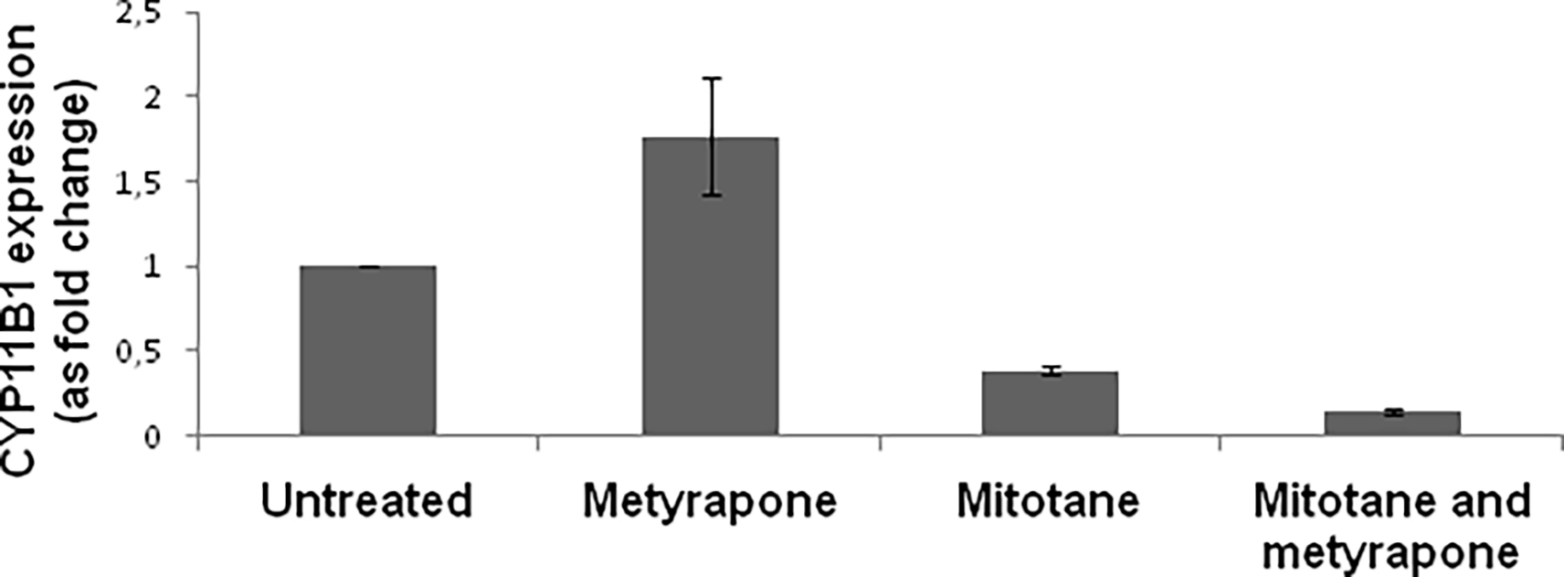
CYP11B1 expression after treatment with mitotane and metyrapone, or both. Data obtained from two independent experiments with two replicates for each experiment, and expressed as fold changes compared to β-actin expression (2-ΔΔCt). A > 2 fold increase was considered significant.

Mitotane treatment induced a strong down-regulation of CYP11B1 gene expression in H295R ACC cell line (< 0.5 fold change compared to untreated cells), while metyrapone induced a non-significant increase (< 2 fold increase compared to untreated cells). Cotreatment with mitotane and metyrapone induced an even greater down-regulation of CYP11B1 gene expression (Fig 4).

### Effects of CYP11B1 inhibition on mitotane activity and metabolization

**Fig 5:**
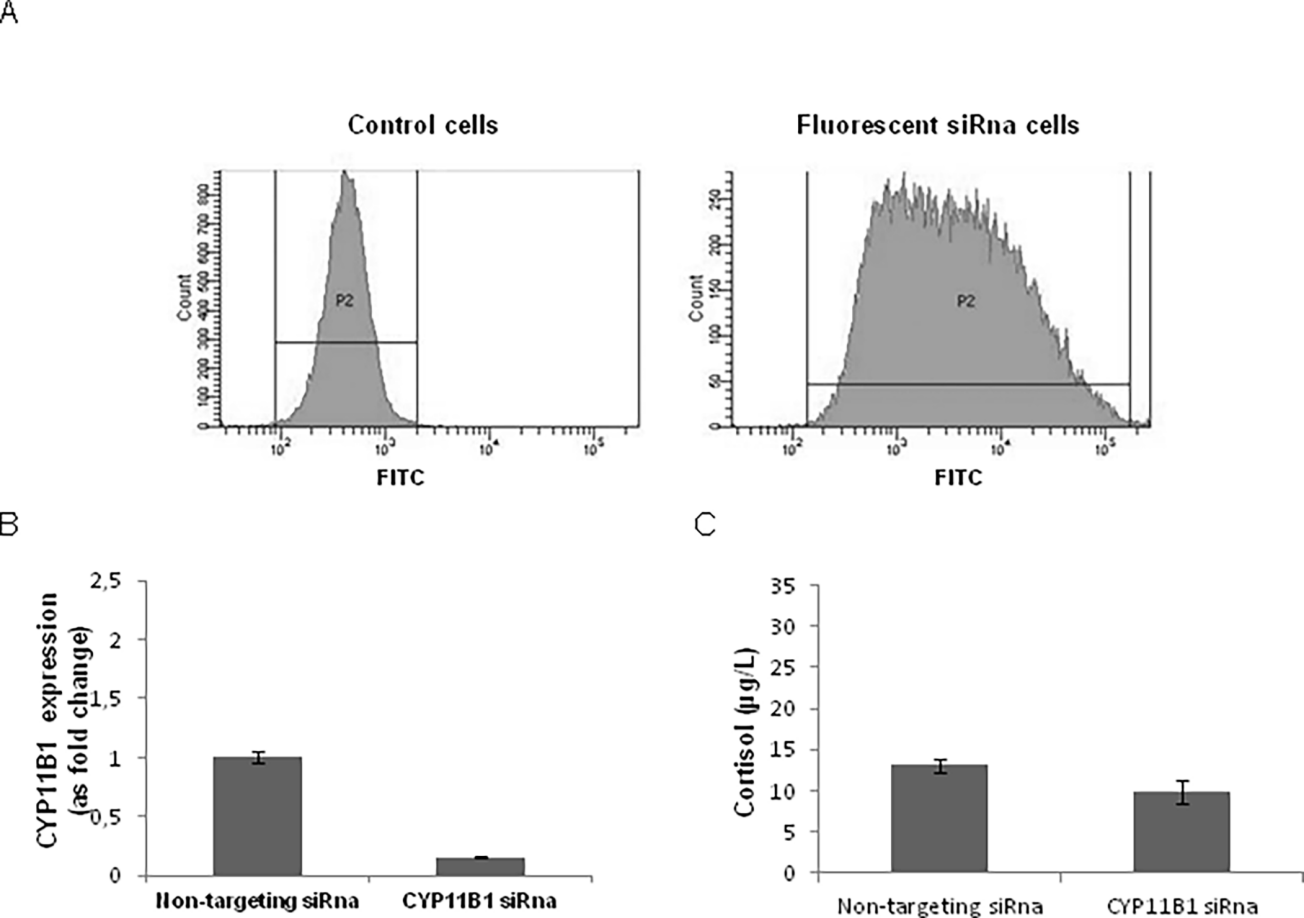
Efficiency of siRna transfection and silencing. (A) H295R cells transfected with a fluorescent siRna indicator. (B) CYP11B1 gene expression in CYP11B1 siRna cells and non-targeting siRna cells. Data obtained from two independent experiments with two replicates for each experiment. (C) Cortisol levels in CYP11B1 siRna cells and non-targeting siRna control cells. Four independent experiments were used to determine each data point.

**Fig 6:**
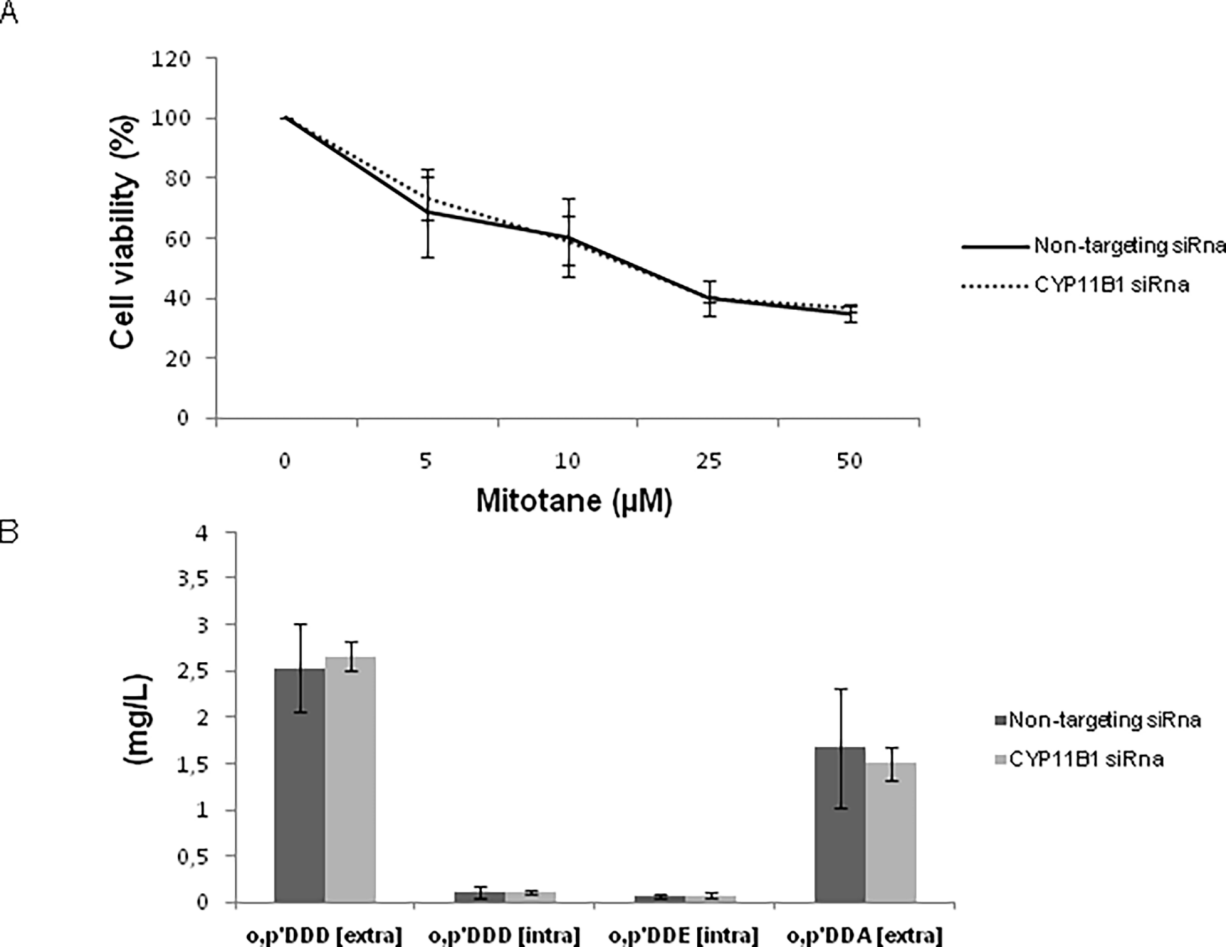
Mitotane cytotoxicity and metabolism after CYP11B1 modulation in H295R cell line. (A) Mitotane cytotoxicity was not influenced by CYP11B1 interference (p = ns, comparing IC50 doses of CYP11B1 siRna cells and non-targeting siRna control cells). Three replicate wells for each experiment (n = 2) were used to determine each data point. (B) CYP11B1 modulation did not influence uptake of mitotane and its metabolization (p = ns, comparing drug levels of CYP11B1 siRna cells and non-targeting siRna control cells). Four independent experiments were used to determinate each data point, measured in HPLC-UV.

To investigate the possible role of CYP11B1 on mitotane activity and metabolism we modulated CYP11B1 expression by RNA specific silencing in H295R cells. Efficiency of transfection was measured by means of flow cytometry showing a 7-fold increase in fluorescent signal in comparison to untransfected control cells (Fig 5A). CYP11B1 mRNA expression in H295R cells was knocked down by CYP11B1 siRna to less than 20% of control cells transfected with non-targeting siRna (Fig 5B). Moreover, knockdown of CYP11B1 was further demonstrated by the reduction of cortisol concentration—cortisol levels for CYP11B1 siRna cells 13. μg/L ± 0.8 vs. non-targeting siRna cells 9.8 μg/L ± 1.3, p = 0.005 - (Fig 5C) (S4 Table). Silencing CYP11B1 gene in H295R cells did not determinate any change in the effects of mitotane on cell viability (Fig 6A) and did not influence mitotane uptake, bioavailability and metabolism into o,p’DDE and o,p’DDA (p = ns) (Fig 6B) (S5 Table).

## Discussion

Mitotane is the reference drug for advanced ACC and clinicians exploit either its cytotoxic effect or the inhibition of different steroidogenic enzymes [1–4, 12]. Mitotane was introduced in clinical use to treat advanced ACC in the sixties [2] but its mechanism of action remains poorly understood. The concept that a metabolic transformation of mitotane is needed for its therapeutic activity is supported by the observation of a huge inter-species variation of the adrenolytic effects of the drug that parallels the different adrenal capability to transform mitotane [11, 12, 16]. It has been suggested that the metabolic transformation of mitotane takes place in the adrenal mitochondria and that CYP11B1 may be involved in the initial hydroxylation of mitotane [6, 13–15, 14]. The fact that CYP11B1 is required to activate mitotane may confer tissue specificity to mitotane, thus explaining why mitotane does not affect SW13 cells [24], which do not express CYP11B1 [25]. However, the concept that mitotane metabolites are important to explicate an anti-tumoral action has been challenged by a recent study, where o,p’-DDA was found to be inactive in H295R cells [26]. Downstream stable metabolites could simply reflect the process of acyl chloride formation. The issue remains controversial since in vitro research by our group found that mitotane metabolites (o,p’-DDE and o,p’-DDA) had cytotoxic activity in H295R cells [27]. Moreover, there is also a study showing that elevated o,p’-DDA levels in patients with advanced ACC were associated with better patient outcome [9].

The present findings do not support the view that CYP11B1 catalyzes a crucial step in the metabolic activation of mitotane, since the concomitant incubation of H295R cells with mitotane and metyrapone, a drug capable of inhibiting CYP11B1 [18], did not influence mitotane-mediated cytotoxic effect. Furthermore, CYP11B1 silencing by specific siRna did not alter mitotane action in our experimental model. Pertinently, changes in CYP11B1 activity or gene expression were also without any major effect on mitotane transformation. In H295R cells, uptake and metabolism of mitotane into o,p’-DDE and o,p’-DDA was unchanged after metyrapone treatment or CYP11B1 silencing.

The concomitant incubation of H295R cells with mitotane and metyrapone resulted in a larger effect on cortisol production. This is consistent with the view that CYP11B1 does not represent a major target in the mitotane-induced inhibition of adrenal steroidogenesis [28], although inhibition of CYP11B1 and 18-hydroxylation (CYP11B2) has been shown in slices of adrenal glands taken from patients treated with mitotane [29]. More recent in vitro studies did not solve the issue whether mitotane is able to consistently affect CYP11B1 [17]. However, the present findings confirm the synergist effect of both drugs to control severe Cushing’s syndrome [19] and are reassuring physicians that the association of metyrapone does not impact on the anti-tumoral efficacy of mitotane. This is clinically relevant since the management of Cushing’s syndrome associated with ACC is often challenging and mitotane has the drawback of a delayed onset of action [2, 12]; thus, the combination with a fast-acting drug like metyrapone may be useful to get a rapid control of severe cortisol excess [30].

To conclude, we have shown that CYP11B1 modulation in H295R cells, by either chemical or molecular inhibition, is not able to affect mitotane action. These data militates against the view that CYP11B1 may play a key role in the metabolic activation of mitotane to ensure its antineoplastic activity. Other mechanisms may explain why mitotane targets the adrenal glands for its cytotoxic action. It has been recently demonstrated how mitotane may inhibit the sterol-O-acyl-transferase 1 (SOAT1) that is predominantly expressed by the adrenals. The inhibition of this enzyme results into an accumulation of free cholesterol at toxic levels for the cell [31]. Conversely, the inhibition of steroidogenesis is not limited to the adrenal glands, since mitotane is able to impair testicular production of testosterone in patients on chronic treatment [32].

## Supporting information

S1 Table Cortisol levels after mitotane and 20 μM metyrapone treatment in H295R cells. Levels are expressed in μg/L.
(TIF)

S2 Table Cell viability ratio levels after mitotane and 20 μM metyrapone treatment in H295R cells. (TIF)

S3 Table Mitotane and metabolites levels after mitotane and 20 μM metyrapone treatment in H295R cells. Levels are expressed in mg/L.
(TIF)

S4 Table Cortisol levels after CYP11B1 silencing in H295R cells. Levels are expressed in μg/L.
(TIF)

S5 Table Mitotane and metabolites levels after CYP11B1 silencing in H295R cells. Levels are expressed in mg/L.
(TIF)

## References

[1] Fassnacht M, Libé R, Kroiss M, Allolio B. Adrenocortical carcinoma: a clinician's update. Nat Rev Endocrinol. 2011;7(6):323–35. doi: 10.1038/nrendo.2010.235 21386792 10.1038/nrendo.2010.235

[2] Terzolo M, Ardito A, Zaggia B, Laino F, Germano A, De Francia S, et al Management of adjuvant mitotane therapy following resection of adrenal cancer. Endocrine. 2012;42(3):521–5. doi: 10.1007/s12020-012-9719-7 22706605 10.1007/s12020-012-9719-7

[3] Terzolo M, Angeli A, Fassnacht M, Daffara F, Tauchmanova L, Conton PA, et al Adjuvant mitotane treatment for adrenocortical carcinoma. N Engl J Med. 2007;356(23):2372–80. doi: 10.1056/NEJMoa063360 17554118 10.1056/NEJMoa063360

[4] Fassnacht M, Terzolo M, Allolio B, Baudin E, Haak H, Berruti A, et al Combination chemotherapy in advanced adrenocortical carcinoma. N Engl J Med. 2012;366(23):2189–97. doi: 10.1056/NEJMoa1200966 22551107 10.1056/NEJMoa1200966

[5] Lehmann TP, Wrzesiński T, Jagodziński PP. The effect of mitotane on viability, steroidogenesis and gene expression in NCI‑H295R adrenocortical cells. Mol Med Rep. 2013;7(3):893–900. doi: 10.3892/mmr.2012.1244 23254310 10.3892/mmr.2012.1244

[6] Cai W, Counsell RE, Schteingart DE, Sinsheimer JE, Vaz AD, Wotring LL. Adrenal proteins bound by a reactive intermediate of mitotane. Cancer Chemother Pharmacol. 1997;39(6):537–40. doi: 10.1007/s002800050610 9118466 10.1007/s002800050610

[7] Hescot S, Slama A, Lombès A, Paci A, Remy H, Leboulleux S, et al Mitotane alters mitochondrial respiratory chain activity by inducing cytochrome c oxidase defect in human adrenocortical cells. Endocr Relat Cancer. 2013;20(3):371– doi: 10.1530/ERC-12-0368 23696597 10.1530/ERC-12-0368

[8] Cai W, Benitez R, Counsell RE, Djanegara T, Schteingart DE, Sinsheimer JE, Wotring LL. Bovine adrenal cortex transformations of mitotane [1-(2-chlorophenyl)-1-(4-chlorophenyl)-2,2-dichloroethane; o,p'-DDD] and its p,p'- and m,p'-isomers. Biochem. Pharmacol. 1995; 17;49(10):1483–9. 7763292 10.1016/0006-2952(95)00028-x

[9] Hermsen IG, Fassnacht M, Terzolo M, Houterman S, den Hartigh J, Leboulleux S, et al Plasma concentrations of o,p'DDD, o,p'DDA, and o,p'DDE as predictors of tumor response to mitotane in adrenocortical carcinoma: results of a retrospective ENS@T multicenter study. J Clin Endocrinol Metab. 2011;96(6):1844–51. doi: 10.1210/jc.2010-2676 21470991 10.1210/jc.2010-2676

[10] Andersen A, Kasperlik-Zaluska AA, Warren DJ. Determination of mitotane (o,p-DDD) and its metabolites o,p-DDA and o,p-DDE in plasma by high-performance liquid chromatography. Ther Drug Monit. 1999;21(3):355–9. 10365653 10.1097/00007691-199906000-00020

[11] Schteingart DE. Adjuvant mitotane therapy of adrenal cancer—use and controversy. N Engl J Med. 2007;356(23):2415–8. doi: 10.1056/NEJMe078087 17554125 10.1056/NEJMe078087

[12] Veytsman I, Nieman L, Fojo T. Management of endocrine manifestations and the use of mitotane as a chemotherapeutic agent for adrenocortical carcinoma. J Clin Oncol. 2009;27(27):4619–29. doi: 10.1200/JCO.2008.17.2775 19667279 10.1200/JCO.2008.17.2775 PMC2754909

[13] Cai W, Counsell RE, Djanegara T, Schteingart DE, Sinsheimer JE, Wotring LL. Metabolic activation and binding of mitotane in adrenal cortex homogenates. J Pharm Sci. 1995;84(2):134–8. 7738789 10.1002/jps.2600840203

[14] Lindhe O, Skogseid B, Brandt I. Cytochrome P450-catalyzed binding of 3-methylsulfonyl-DDE and o,p'-DDD in human adrenal zona fasciculata/reticularis. J Clin Endocrinol Metab. 2002;87(3):1319–26. doi: 10.1210/jcem.87.3.8281 11889204 10.1210/jcem.87.3.8281

[15] Lund BO, Lund J. Novel involvement of a mitochondrial steroid hydroxylase (P450c11) in xenobiotic metabolism. J Biol Chem. 1995;270(36):20895–7. 7673111 10.1074/jbc.270.36.20895

[16] Waszut U, Szyszka P, Dworakowska D. Understanding mitotane mode of action. J Physiol Pharmacol. 2017;68(1):13–26. 28456766

[17] Lin CW, Chang YH, Pu HF. Mitotane exhibits dual effects on steroidogenic enzymes gene transcription under basal and cAMP-stimulating microenvironments in NCI-H295 cells. Toxicology. 2012;298(1–3):14–23. doi: 10.1016/j.tox.2012.04.007 22546480 10.1016/j.tox.2012.04.007

[18] Daniel E, Newell-Price JD. Therapy of endocrine disease: steroidogenesis enzyme inhibitors in Cushing's syndrome. Eur J Endocrinol. 2015;172(6):R263–80. doi: 10.1530/EJE-14-1014 25637072 10.1530/EJE-14-1014

[19] Kamenický P, Droumaguet C, Salenave S, Blanchard A, Jublanc C, Gautier JF, et al Mitotane, metyrapone, and ketoconazole combination therapy as an alternative to rescue adrenalectomy for severe ACTH-dependent Cushing's syndrome. J Clin Endocrinol Metab. 2011;96(9):2796–804. doi: 10.1210/jc.2011-0536 21752886 10.1210/jc.2011-0536

[20] Owen LJ, Halsall DJ, Keevil BG. Cortisol measurement in patients receiving metyrapone therapy. Ann Clin Biochem. 2010;47(Pt 6):573–5. doi: 10.1258/acb.2010.010167 20926474 10.1258/acb.2010.010167

[21] Hermansson V, Asp V, Bergman A, Bergström U, Brandt I. Comparative CYP-dependent binding of the adrenocortical toxicants 3-methylsulfonyl-DDE and o,p'-DDD in Y-1 adrenal cells. Arch Toxicol. 2007;81(11):793–801. doi: 10.1007/s00204-007-0206-5 17487473 10.1007/s00204-007-0206-5

[22] Breen M, Breen MS, Terasaki N, Yamazaki M, Lloyd AL, Conolly RB. Mechanistic computational model of steroidogenesis in H295R cells: role of oxysterols and cell proliferation to improve predictability of biochemical response to endocrine active chemical—metyrapone. Toxicol Sci. 2011;123(1):80–93. doi: 10.1093/toxsci/kfr167 21725065 10.1093/toxsci/kfr167

[23] De Francia S, Pirro E, Zappia F, De Martino F, Sprio AE, Daffara F, et al A new simple HPLC method for measuring mitotane and its two principal metabolites Tests in animals and mitotane-treated patients. J Chromatogr B Analyt Technol Biomed Life Sci. 2006;837(1–2):69–75. doi: 10.1016/j.jchromb.2006.04.005 16698327 10.1016/j.jchromb.2006.04.005

[24] Volante M, Terzolo M, Fassnacht M, Rapa I, Germano A, Sbiera S, et al Ribonucleotide reductase large subunit (RRM1) gene expression may predict efficacy of adjuvant mitotane in adrenocortical cancer. Clin Cancer Res. 2012;18(12):3452–61. doi: 10.1158/1078-0432.CCR-11-2692 22547773 10.1158/1078-0432.CCR-11-2692

[25] Kroiss M, Reuss M, Kühner D, Johanssen S, Beyer M, Zink M, et al Sunitinib Inhibits Cell Proliferation and Alters Steroidogenesis by Down-Regulation of HSD3B2 in Adrenocortical Carcinoma Cells. Front Endocrinol (Lausanne). 2011;2:27. 22654799 10.3389/fendo.2011.00027 PMC3356136

[26] Hescot S, Paci A, Seck A, Slama A, Viengchareun S, Trabado S, et al The lack of antitumor effects of o,p'DDA excludes its role as an active metabolite of mitotane for adrenocortical carcinoma treatment. Horm Cancer. 2014;5(5):312–23. doi: 10.1007/s12672-014-0189-7 25026941 10.1007/s12672-014-0189-7 PMC5127823

[27] Germano A, Rapa I, Volante M, De Francia S, Migliore C, Berruti A, et al RRM1 modulates mitotane activity in adrenal cancer cells interfering with its metabolization. Mol Cell Endocrinol. 2015;401:105–10. doi: 10.1016/j.mce.2014.11.027 25497672 10.1016/j.mce.2014.11.027

[28] Young RB, Bryson MJ, Sweat ML, Street JC. Complexing of DDT and o,p'DDD with adrenal cytochrome P-450 hydroxylating systems. J Steroid Biochem. 1973;4(6):585–91. 4789318 10.1016/0022-4731(73)90033-2

[29] Touitou Y, Bogdan A, Luton JP. Changes in corticosteroid synthesis of the human adrenal cortex in vitro, induced by treatment with o,p'-DDD for Cushing's syndrome: evidence for the sites of action of the drug. J Steroid Biochem. 1978;9(12):1217–24. 745418 10.1016/0022-4731(78)90015-8

[30] Claps M, Cerri S, Grisanti S, Lazzari B, Ferrari V, Roca E, et al Adding metyrapone to chemotherapy plus mitotane for Cushing's syndrome due to advanced adrenocortical carcinoma. Endocrine. 2017. 10.1007/s12020-017-1428-9 29019062

[31] Sbiera S, Leich E, Liebisch G, Sbiera I, Schirbel A, Wiemer L, et al Mitotane Inhibits Sterol-O-Acyl Transferase 1 Triggering Lipid-Mediated Endoplasmic Reticulum Stress and Apoptosis in Adrenocortical Carcinoma Cells. Endocrinology. 2015;156(11):3895–908. doi: 10.1210/en.2015-1367 26305886 10.1210/en.2015-1367

[32] Daffara F, De Francia S, Reimondo G, Zaggia B, Aroasio E, Porpiglia F, et al Prospective evaluation of mitotane toxicity in adrenocortical cancer patients treated adjuvantly. Endocr Relat Cancer. 2008;15(4):1043–53. doi: 10.1677/ERC-08-0103 18824557 10.1677/ERC-08-0103

