# Supplementary figures and images for: CYP11B1 has no role in mitotane action and metabolism in adrenocortical carcinoma cells
Source: PLoS One. 2018 May 7;13(5):e0196931. doi: 10.1371/journal.pone.0196931 (PMC5937768; doi:10.1371/journal.pone.0196931)

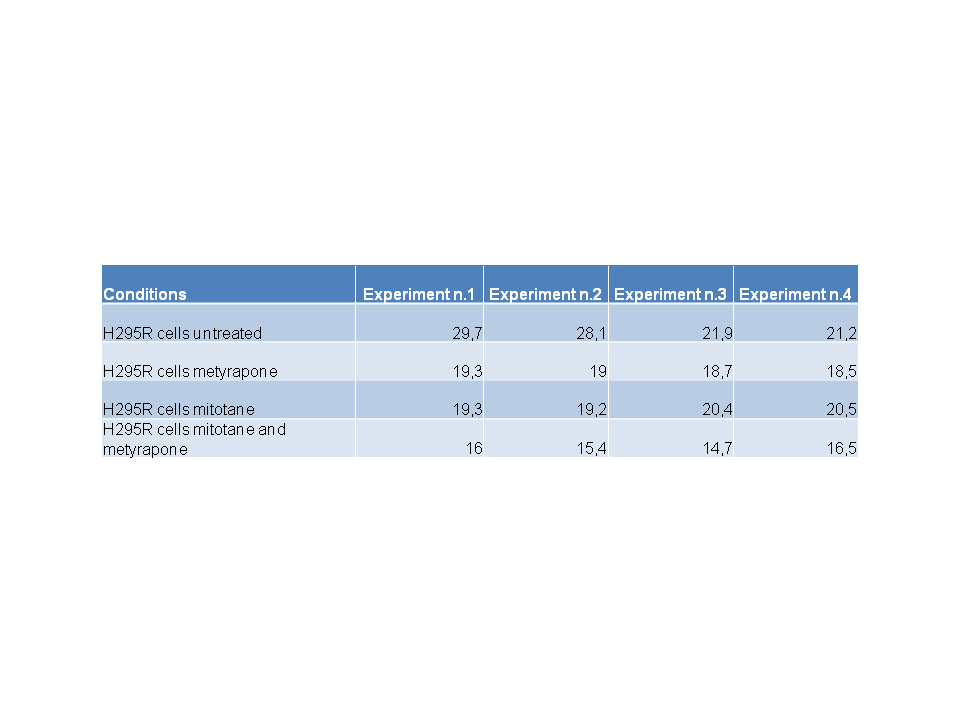

Supplement: S1 Table — Levels are expressed in μg/L. (TIF) [file pone.0196931.s001.tif]

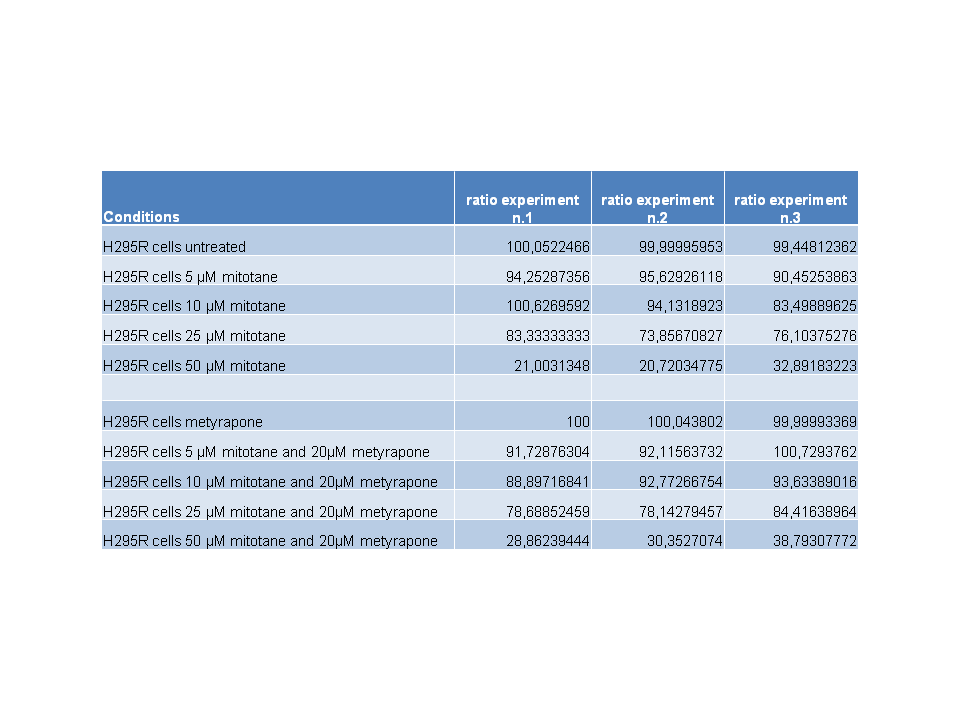

Supplement: S2 Table — (TIF) [file pone.0196931.s002.tif]

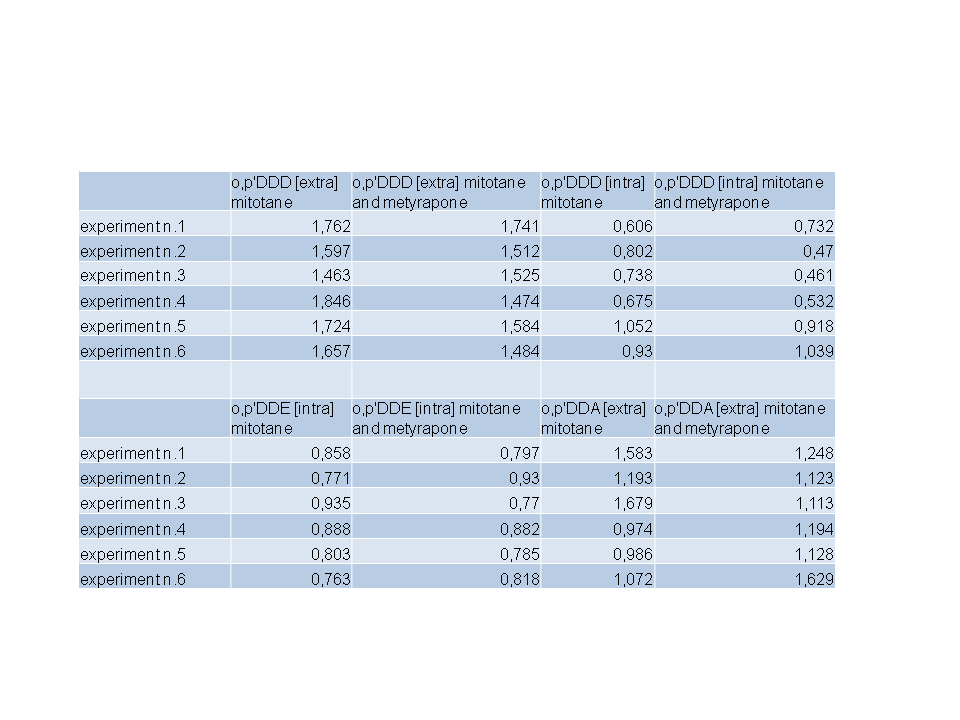

Supplement: S3 Table — Levels are expressed in mg/L. (TIF) [file pone.0196931.s003.tif]

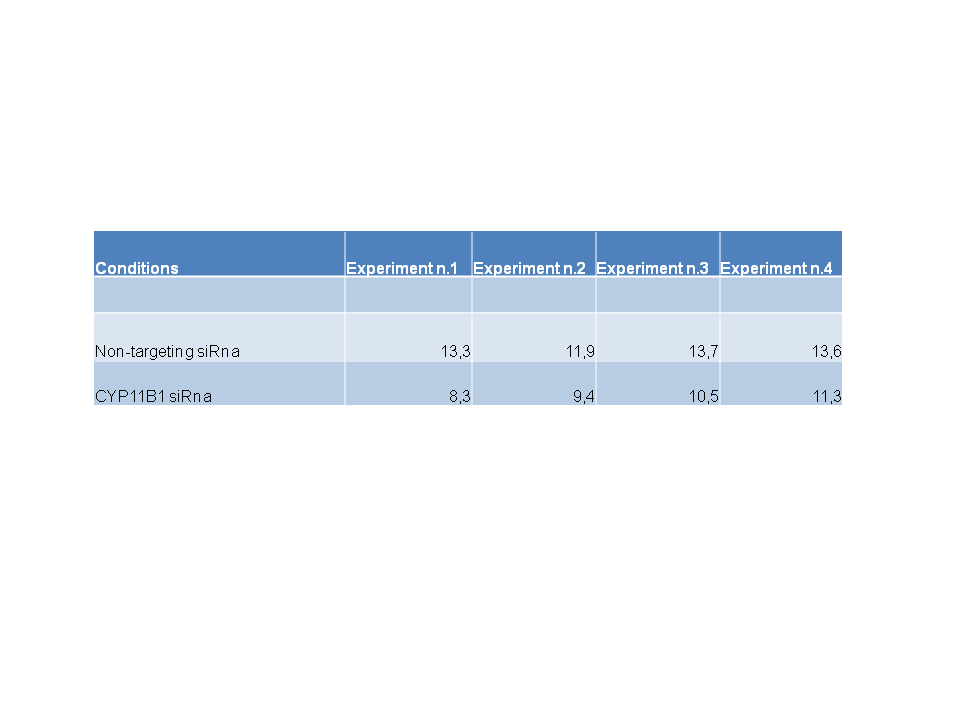

Supplement: S4 Table — Levels are expressed in μg/L. (TIF) [file pone.0196931.s004.tif]

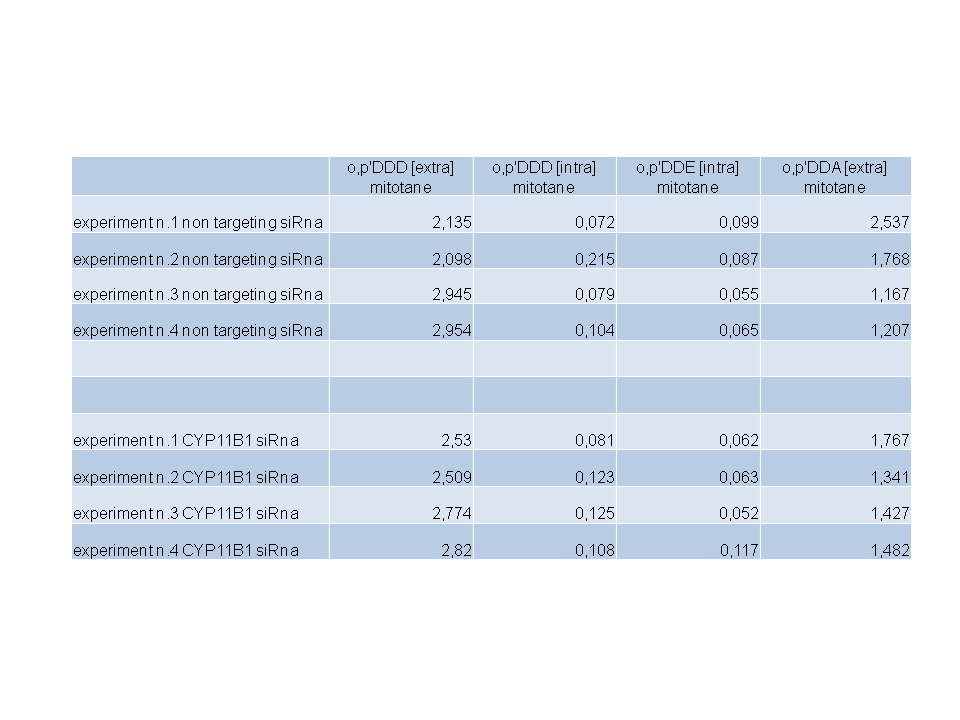

Supplement: S5 Table — Levels are expressed in mg/L. (TIF) [file pone.0196931.s005.tif]
